# Homogenized Elastic Properties of Graphene for Small Deformations

**DOI:** 10.3390/ma6093764

**Published:** 2013-09-03

**Authors:** Eduard Marenić, Adnan Ibrahimbegovic, Jurica Sorić, Pierre-Alain Guidault

**Affiliations:** 1 LMT-Cachan (ENS-Cachan/CNRS/UPMC/PRES UniverSud Paris) 61, avenue du Président Wilson, Cachan F-94235, France; E-Mails: adnan.ibrahimbegovic@ens-cachan.fr (A.I.); guidault@lmt.ens-cachan.fr (P.-A.G.); 2 Faculty of Mechanical Engineering and Naval Architecture, University of Zagreb, Ivana Lucica 5, Zagreb 10000, Croatia; E-Mail: jurica.soric@fsb.hr

**Keywords:** graphene, elastic properties, molecular mechanics, stiffness bounds

## Abstract

In this paper, we provide the quantification of the linear and non-linear elastic mechanical properties of graphene based upon the judicious combination of molecular mechanics simulation results and homogenization methods. We clarify the influence on computed results by the main model features, such as specimen size, chirality of microstructure, the effect of chosen boundary conditions (imposed displacement *versus* force) and the corresponding plane stress transformation. The proposed approach is capable of explaining the scatter of the results for computed stresses, energy and stiffness and provides the bounds on graphene elastic properties, which are quite important in modeling and simulation of the virtual experiments on graphene-based devices.

## 1. Introduction

Graphene is the name given to a single atomic layer of carbon atoms tightly packed into a two-dimensional (2D) honeycomb lattice. It is also a basic building block for carbon nano-structures of other dimensionality, since it can be wrapped up to form fullerenes, rolled into nanotubes or stacked into graphite. In other words, it is the building block of several carbon allotropes. Studies of graphene have been going on for more than fifty years. Nowadays, it is widely used for describing properties of various carbon-based materials. Although known as an integral part of carbon-based materials, graphene was initially presumed not to exist in the free state. Actually, in the early 1940s, scientists claimed that strictly 2D crystals could not exist, since they were thermodynamically unstable. Thus, graphene was usually described as an “academic” material that was believed to be unstable in the formation of “curved” structures, such as fullerene and carbon nanotube (CNT). When free-standing graphene was found in 2004 [1], the model of the single-layer graphene sheet (SLGS) became interesting again [2]. Moreover, the possibility to grow the graphene in larger sizes, required for industrial applications, prompted a renewed interest in investigation, simulation and testing, seeking to improve the understanding of 2D crystals, like graphene. The experimental measurement of the mechanical properties of nano-structured materials is still considered a difficult task that requires tests to be performed at the nano-scale. There is not yet a large number of existing works on experimental evaluation of the mechanical properties of graphene (e.g., see [3,4]). Consequently, quantifying the elastic properties by numerical simulations becomes of even greater importance. A numerical simulation of this kind ought to start at the microscale. A major feature of the graphene structure is the hexagon pattern, which repeats itself periodically in the plane. As the result of periodicity, each atom is bonded to three neighboring atoms. Each atom in the planar, hexagonal structure is bonded to three neighboring atoms, apart from the boundary atoms, which only have two neighbors. This microstructure is the result of the process of sp${}^{2}$ hybridization, during which one s-orbital and two p-orbitals combine to form three hybrid sp${}^{2}$-orbitals at $120^{\circ }$ to each other within a plane [5,6]. This covalent bond, referred to as the *σ*-bond, is a strong chemical bond and plays an important role in providing the impressive mechanical properties of carbon nano-structures, giving a Young’s modulus close to 1000 GPa and a tensile strength of around 100 GPa.

The practical application of graphene with its exceptional mechanical, thermal and electrical properties are yet to be discovered and flexible electronics, ultra-performance batteries, reinforcements in advanced composites and building blocks for nano-electro-mechanical systems (NEMS) are just some of the examples [8]. For instance, graphene has extremely low mass density and high mechanical strength, which are key qualities for efficient wide-frequency-response electrostatic audio speaker design. As shown recently in [7], a speaker/earphone with a graphene diaphragm has excellent frequency response across the entire audio frequency range and with performance matching or surpassing commercially available products. For potential applications of graphene materials, such as reinforcement agents to strengthen composites or structural parts in nano-electro-mechanical systems (NEMS) devices, the mechanical response of the graphene under different loading programs and boundary conditions should still be better understood. One of the fundamental issues that scientists and engineers are confronted with is the characterization of the mechanical behavior of single-layer graphene sheet (SLGS). All the difficulties mentioned regarding experimental measurements and, also, the need for an effective design tool for novel applications have encouraged the development of computer simulation methods that can accurately predict the behavior of this promising material. The main focus of work concerns the isotropic, linear elastic mechanical behavior, characterized by the predicted value of Young’s modulus (*E*) and Poisson’s ratio (*ν*). The vast majority of previously proposed formulations and computational methods leads to radically different results regarding graphene elastic properties. We present in this paper a review of some recent research and the results of simulations, and more importantly, we identify the main mechanisms resulting in such a large dispersion of elastic properties.

A large scatter of the Young’s modulus value was mentioned for the first time in [9]. The same work also gave a molecular mechanics (MM) study of two initial configurations, with and without equilibrium adjustment of atoms before the loading process. The main conclusion was that the computed values fit in two groups: first, the values of *E* around 700 GPa and, then, those of around 1000 GPa. These correspond, respectively, to the minimized (equilibrium) and unminimized (with no potential minimization) configuration. The interatomic potential used therein is the Tersoff-Brenner [10], defined as the pair potential with the addition of a cut-off function and multibody parameter (see, also, [11]). This potential when minimized (*i.e.*, solved for unload configuration) yields a slightly different configuration than the initial formed of regular hexagonal structure. This effect is due to coordination number, *i.e.*, the different number of neighboring atoms on the boundary, and is noticeable near the boundary. The bond length of interior bonds in the finite graphene is already close to that of infinite graphene (yet, referred to as bulk). They conclude that the minimization of potential is one of the reasons for Young’s modulus scatter. The prescribed displacement is used on the edges of rather small graphene sample consisting of 120 atoms (around 1.5 nm × 1.5 nm). The results are in good agreement with the one presented in [12]. In [9], they also consider two models in the analysis, with only two or all four edges constrained to be straight. To our knowledge, this is the only research that includes the influence of boundary conditions on the elastic properties of graphene, even though it is by no means systematic. In [13], they use tight-binding (TB) (reported E=910 GPa) and the molecular dynamics (MD) method (reported E=1010±30 GPa) with reactive bond-order (REBO) potential to study mechanical properties of graphene, *i.e.*, stripes of graphene called graphene nanoribbon (GNR). They perform molecular dynamics (MD) uniaxial tensile tests under deformation-control with periodic boundary conditions to study the chirality effects on bulk (infinite size) graphene or force-control to study size and chirality effects of graphene nanoribbon (GNR) (finite). They show the convergence of *E* with the size of the GNR and the influence of the chirality (armchair *versus* zigzag) on the computed value of Young’s modulus. The results are in reasonable agreement with experiments, which report E=1000±100 GPa (see [4]) and *ab initio* (see [14,15]). Similar tension analysis using MD is done by Xu [16], with emphasis on the dynamical effects on fracture. Lu *et al*. [17,18] pointed out the effect of edge structures on the mechanical behavior of GNRs. Particularly, they focus on the nonlinear behavior of GNRs under quasi-static uniaxial tension using molecular mechanics (MM), emphasizing the effects of armchair and zigzag edges, without and with hydrogen passivation, on elastic modulus and fracture. They report Youngs modulus to be 714 GPa using the REBO potential. Another interesting strategy to model nanostructures, introduced in [19], is based on the so-called equivalent atomistic continuum-structural mechanics approach. In this approach, typical finite elements of structural mechanics, such as bar, beam and shell, are used with appropriate mechanical properties to simulate the behavior of graphene layers and carbon nanotubes. An extension of the truss-lattice (FEM) model from [19] is proposed in [20], where the equivalent atomistic continuum-structural mechanics approach is combined with the theory of cellular solids micromechanics. The AMBER and Morse interatomic potentials are used, and closed form solutions for the in-plane elastic properties of single-layer graphene sheet (SLGS) are given. In [21], a structural mechanics approach was used based upon nonlinear spring finite element (FE) to simulate the SLGS behavior, represented by a modified Morse potential. The later approach is used in [22] to show how size and chirality influence mechanical properties of SLGS. Besides the mentioned study, an exhaustive literature review of the mechanical properties of SLGS is presented in [22], separating them into three groups. The first group is related to the use of the MD method, for which Young’s modulus remains in the range *E* = 710–1200 GPa. The MM *i.e.*, structural mechanics methods, forms the second group and ranges from 940 to 5510 GPa. Finally, for the experimental methods, they report a range of *E* = 700–7000 GPa. However, the modulus of E=7000 GPa considers the thickness of 0.075 nm, while for the rest of the studies, it considers mostly around 0.34 nm; see [22] and the references therein. The dispersion of the mechanical properties of carbon nanostructures attributed to the uncertainty related to the thickness of the nanostructure is known as Yakobson’s paradox [23]. Most of the atomistic calculations agree on the numerical value of product E·t of Young’s modulus (*E*) and thickness (*t*). There are cases where there is no need to know *E*. However, if a specific value is needed, then an estimate of *t* is required to compute it. If a thickness equivalent to that of graphite interlayer spacing, around 0.34 nm, is assumed, *E* turns out to be roughly 1 TPa. In the case of the shell model, both the tension and bending rigidity needs to be calculated in order to obtain the thickness. In this case, the elastic modulus results in an estimate of 5–6 TPa. In [23], this issue is addressed, and a resolution is provided by relating the relevant rigidities analytically to the interatomic potential. In [24], the results are mostly repeated from [22] for pristine SLGS, with emphasis on the influence of the circular defect on the elastic mechanical properties of graphene. There is also a number of papers covering the modeling of the graphene using a theoretical framework of the nonlinear continuum mechanics in combination with the interatomic potential(e.g., [25,26]). A rigorous homogenization technique has been also developed by Caillerie *et al*. [27] o calculate stress tensors, in terms of, first, Piola-Kirchhoff and Cauchy, considering stretching and bond angle variation. The later approaches are really effective, especially when combined with finite element method (FEM); however, they do not allow the simulation of defects in graphene.

We give a brief summary of the mechanisms causing the discrepancy presented above. The reason for the results scatter, obtained by different simulations, is first of all related to the formulation differences. This concerns MM, MD, continuum mechanics and the *ab initio* methods mentioned above. Each of these methods has known advantages and disadvantages and leads the differences in the elastic response of SLGS. The results discrepancy is partly attributed to a particular choice of intearatomic potential that drives the atomic system. Namely, while for SLGS, Tersoff-Brenner-like potential is usually the first choice, Morse, AMBER and second generation REBO potentials are also used. Furthermore, the dispersion of the equivalent mechanical properties of SLGS is related to the above-mentioned uncertainty of the thickness. Apart from these general reasons related more to the formulation of simulation method, there is a number of other mechanisms responsible for the scatter. They are related to size effects, relaxation (minimization of the energy due to coordination), chirality and edge passivation. The size effects results in size-dependent mechanical properties. Based on that observation, it is suggested that comparisons of results should be performed between graphene specimens of the same size. This mostly applies for sizes below 10 nm. The chirality is related to the intrinsic hexagonal structure and its orientation with respect to the load, while edge passivation concerns the boundary effects.

In the previous works dealing with simulation of SLGS, the elastic modulus is calculated via average results for the stress and strain as the corresponding fit to the strain energy value. The latter is, in general, obtained from atomistic simulation. An alternative procedure to obtain the elastic properties is by averaging or homogenization of the discrete model. The latter can provide the homogenization bounds for the stiffness (see [28]), by making the appropriate choice of boundary conditions. This particular point, to our knowledge, has not been discussed when it comes to the elastic properties of the SLGS. More precisely, we exploit the concept of apparent properties, first introduced in [29], where the hierarchy of bounds was established for the effective properties for the homogeneous boundary conditions. We perform numerical tests to establish those bounds, in a similar manner to the one proposed in [28], but in the context of the MM of graphene. More precisely, in this paper, we use MM modeling and simulation to capture the influence of the imposed boundary conditions (displacement or force) on elastic properties. In particular, by following the theoretical predictions in [29], we can establish that the linear elastic stiffness obeys the following order of bounds: $C_{s}^{app}\leq C^{eff}\leq C_{d}^{app}$; here $C_{s}^{app}$ denotes the apparent stiffness obtained with homogeneous traction boundary conditions and $C_{d}^{app}$ is the one obtained with homogeneous displacement boundary conditions. An equivalent procedure can be used for comparison between the computational (virtual) experiments on graphene *versus* the real experimental measurements in load or displacement control in both a linear and non-linear regime. A procedure of this kind is of direct interest for the development of integrated graphene-based devices.

The paper is organized as follows. In the following section, we introduce the numerical model, defining first the microstructure of graphene and the corresponding force field in agreement with the chosen interatomic potential. Next, the boundary conditions and computational procedures for the tensile tests are described. In Section 3, we present the results of our numerical computations, along with a detailed discussion of their interpretations. The concluding remarks are stated in Section 4.

## 2. Graphene Model Properties and Homogenization Procedure

### 2.1. Problem Statement

We consider a domain, Ω, in a three-dimensional Euclidean space, $R^{3}$, which is occupied by *N* atoms placed within graphene microstructure. Let $X_{i}$ and $x_{i}$ denote, respectively, the position vectors in the reference and the current configurations of atom *i*, where i=1,…,N. The corresponding displacement vector of atom *i* can be defined by $d_{i}=x_{i}-X_{i}$. The boundary conditions ought to be defined atom-wise, such that either the displacement, ${\bar{d}}_{i}$, or the external point force, ${\bar{f}}_{i}$, takes an imposed value. These conditions are imposed in a quasi-static manner, with the corresponding incremental sequence. The total energy, $E_{tot}$, stored in the atomic microstructure is given by:

(1) $$E_{tot}=U\left(x_{1},\ldots ,x_{N}\right)-\sum_{i}^{N}{\bar{f}}_{i}\cdot d_{i}$$

where *U* denotes the energy stored in the atomic bonds, as presented in sequel, and the second term on the right-hand side represents the external energy, $E^{ext}$. The state of equilibrium of the atomistic system requires the variation of the total energy to be equal to zero:

(2) $$\delta E_{tot}=\sum_{i}^{N}\left(\frac{\partial U}{\partial x_{i}}-{\bar{f}}_{i}\right)\cdot \delta x_{i}=0$$

where $\delta x_{i}$ represents the kinematically admissible virtual motion. Linearizing Equation (2) and writing the result in matrix notation leads to:

(3) $$K^{\left(k\right)}\Delta d^{\left(k\right)}=F^{\left(k\right)}$$

where $\Delta d^{\left(k\right)}$ is the displacement increment corresponding to the *k*-th load increment, whereas $K^{\left(k\right)}$ and $F^{\left(k\right)}$ are global stiffness and the residual vector, respectively. The latter can explicitly be defined as:

(4) $$K_{ij}=\frac{\partial^{2}U}{\partial x_{i}\partial x_{j}},\;F_{i}=\frac{\partial U}{\partial x_{i}}-{\bar{f}}_{i}$$

Unlike conventional FEM for continuum mechanics (e.g., [30]), we derive and assemble the stiffness and residual matrices by looping over all atoms. In this manner, we can account for the corresponding pairwise and angular potentials defined for each atom interacting with its neighbors. This kind of procedure (e.g., [31]) is further discussed in detail. We first compute the first and second derivatives of $E_{tot}$, as needed in governing Equation (4). Due to the non-linear nature of the interatomic potentials (as shown in Section 2.2) and geometrically nonlinear kinematics, we need to use an incremental-iterative solver. For each load increment, several Newton iterations are performed, until convergence criteria are met in terms of an energy test, which checks both the residual force and incremental displacement. At each iteration, (k), the atomic positions are updated as follows:

(5) $$x_{i}^{\left(k+1\right)}=x_{i}^{\left(k\right)}+\Delta d^{\left(k\right)}$$

The initial iteration, (k)=0, starts at the initial configuration of the atomic system, with the position vector, $x_{i}^{\left(0\right)}=X_{i}$. The procedure is terminated when the convergence is achieved for the last load increment.

### 2.2. Choice of Interatomic Potential

The interatomic potential is assumed to be an at least twice continuously differentiable function, which ensures that the stiffness matrix, K, in Equation (4) is defined at each deformed configuration. Moreover, according to the given definition of K in Equation (4), this matrix is symmetric. For the atomistic simulation of a graphene sheet, a modified Morse potential is used [32]. The chosen potential consists of pair and angular parts:

(6) $$U=U_{p}\left(r\right)+U_{\theta }\left(\theta \right)$$

The first term is a function of the chosen atom distance, *r*, to its first neighbor, whereas the second depends upon angle *θ* between particular atom bonds. Thus, for atom *i*, $r=∥r_{ij}∥,\;r_{ij}=x_{i}-x_{j}$ is its distance to neighbor *j*, and *θ* is the current angle between the bonds, i-j and i-k. The energy of the pair or angular part is given by summing up the energies of the bonds, $V_{p}^{M}$ and $V_{\theta }^{M}$:

(7) $$U_{p}\left(r\right)=\sum_{\mathrm{bonds}}V_{p}^{M},\;U_{\theta }\left(\theta \right)=\sum_{\mathrm{angles}}V_{\theta }^{M}$$

**Figure 1:**
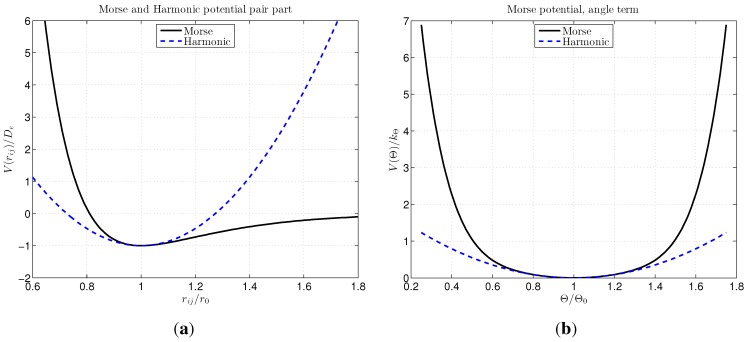
Distribution of the Morse potential and equivalent harmonic potential for the pair part (**a**) and for the angular part (**b**).

For the modified Morse potential, the bond energy terms are given as:

(8) $$V_{p}^{M}\left(r\right)=D_{e}\left[{\left(1-e^{-\beta (r-r_{0})}\right)}^{2}-1\right]$$

(9) $$V_{\theta }^{M}\left(\theta \right)=\frac{1}{2}k_{\theta }{\left(\theta -\theta_{0}\right)}^{2}\left[1+k_{sext}{\left(\theta -\theta_{0}\right)}^{4}\right]$$

where the constants of the potential (e.g., [32]) are: $D_{e}=6.03105\times 10^{-19}$ Nm, $\beta =2.625\times 10^{10}$ m${}^{-1}$, $k_{\theta }=0.9\times 10^{-18}$ Nm rad${}^{-2}$, $k_{sext}=0.754$ rad${}^{-4}$, the initial value of the bond length, $r_{0}=∥R_{ij}∥=1.39\times 10^{-10}$ m, and the bond angle, $\theta_{0}=2\pi /3$ rad. For the small displacement case with ∥∇d∥≪1, the Morse potential can be replaced by a computationally cheaper interatomic potential of quadratic form, the so-called harmonic potential:

(10) $$V_{p}^{H}\left(r\right)=\frac{1}{2}k_{p}^{H}{\left(r-r_{0}\right)}^{2}$$

(11) $$V_{\theta }^{H}=\frac{1}{2}k_{\theta }{\left(\theta -\theta_{0}\right)}^{2}$$

where the parameter, $k_{p}^{H}=2\beta^{2}D_{e}$, was obtained by the fit and $k_{\theta }$ has the same value as for the Morse model. The plots of the mentioned expressions for both Morse and the harmonic potential are shown in Figure 1.

We present a detailed derivation of the residual force and tangent stiffness matrices related to the chosen atom in Equation (4). These results can be obtained directly from the defined strain energy by the interatomic potential, $U_{p}\left(r\right)$ and $U_{\theta }\left(\theta \right)$*i.e.*, bond energies, $V_{p}\left(r\right)$ and $V_{\theta }\left(\theta \right)$. For example, the internal force, $P_{i}$, on atom *i*, due to pair interaction in the bond, i-j, can be written as:

(12) $$P_{i}=\frac{\partial U_{p}}{\partial d_{i}}=\frac{\partial V_{p}\left(r\right)}{\partial d_{i}}=\frac{\partial V_{p}}{\partial r}\frac{\partial r}{\partial r_{ij}}\frac{\partial r_{ij}}{\partial d_{i}}$$

In the last equation, we consider that $r_{ij}=d_{j}-d_{i}+R_{ij}$, and derivatives, $\frac{\partial r}{\partial r_{ij}}=\frac{r_{ij}}{r}$. Additionally, $\frac{\partial r_{ij}}{\partial d_{i}}=-1$. Analogously, the internal force on atom *j* from the pair potential is given as:

(13) $$P_{j}=\frac{\partial U_{p}}{\partial d_{j}}=\frac{\partial V_{p}\left(r\right)}{\partial d_{j}}$$

Using the vector notation for internal forces of pair potential, $P_{i-j}={\left[P_{i}\;P_{j}\right]}^{T}$, the global internal force can be obtained through the assembly process:

(14) $$P_{p}=A_{i,j}^{n_{p}}P_{i-j}$$

where A denotes the assembly operator and $n_{p}$, the number of pair bonds. For the angle part of the potential, the generalized internal force can be written as:

(15) $$P_{i}^{\theta }=\frac{\partial U_{\theta }}{\partial d_{i}}=\frac{\partial V_{\theta }\left(\theta \right)}{\partial d_{i}}=\frac{\partial V_{\theta }}{\partial \theta_{jik}}\frac{\partial \theta_{jik}}{\partial \cos\theta_{jik}}\frac{\partial \cos\theta_{jik}}{\partial d_{i}}$$

where we would need the following results:

(16) $$\begin{array}{cc} \frac{\partial \theta_{jik}}{\partial \cos\theta_{jik}} & =-\csc\theta_{jik}=-\frac{1}{\sin\theta_{jik}},\;\theta_{jik}\neq 0+k\pi \end{array}$$

(17) $$\begin{array}{cc} \cos\theta_{jik} & =\frac{r_{ij}\cdot r_{ik}}{\left|r_{ij}\right|\left|r_{ik}\right|} \end{array}$$

(18) $$\begin{array}{cc} \frac{\partial \cos\theta_{jik}}{\partial d_{i}} & =\frac{\partial \cos\theta_{jik}}{\partial r_{ij}}\frac{\partial r_{ij}}{\partial d_{i}}+\frac{\partial \cos\theta_{jik}}{\partial r_{ik}}\frac{\partial r_{ik}}{\partial d_{i}} \end{array}$$

A similar procedure is followed to obtain $P_{j}^{\theta }$ and $P_{k}^{\theta }$ using the relations:

(19) $$P_{j}^{\theta }=\frac{\partial U_{\theta }}{\partial d_{j}},\;P_{k}^{\theta }=\frac{\partial U_{\theta }}{\partial d_{k}}$$

Denoting the vector of internal forces for angle potential, $P_{i-j-k}={\left[P_{i}^{\theta }\;P_{j}^{\theta }\;P_{k}^{\theta }\right]}^{T}$, the corresponding global generalized internal force pertinent to angle change is obtained by global assembly, which may be expressed as:

(20) $$P_{\theta }=A_{i,j,k}^{n_{\theta }}P_{i-j-k}$$

The tangent stiffness matrix associated with the pair part of potential can then be written in the form:

(21) $$K_{i-j}=\left[\begin{array}{cc} P_{i,i} & P_{i,j}\\ P_{j,i} & P_{j,j} \end{array}\right]$$

where $P_{i,j}=\frac{\partial P_{i}}{\partial d_{j}}$. Similarly, the tangent stiffness matrix associated with the angle part of the potential (*i.e.*, angle $\theta_{jik}$) is defined as:

(22) $$K_{i-j-k}=\left[\begin{array}{ccc} P_{i,i}^{\theta } & P_{i,j}^{\theta } & P_{i,k}^{\theta }\\ P_{j,i}^{\theta } & P_{j,j}^{\theta } & P_{j,k}^{\theta }\\ P_{k,i}^{\theta } & P_{k,j}^{\theta } & P_{k,k}^{\theta } \end{array}\right]$$

The assembly procedure for the stiffness matrix is performed again, in order to take into account all contributions from pair and angle bonds:

(23) $$K_{p}=A_{i,j}^{n_{p}}K_{i-j},\;K_{\theta }=A_{i,j,k}^{n_{\theta }}K_{i-j-k}$$

It has been noted before ([33,34]) that an assembly procedure of this kind can be carried out pretty much in the same manner as the standard FEM assembly (e.g., [30]), thus resulting in the model that fits within the standard computer code architecture.

### 2.3. Choice of Boundary Conditions and Computational Procedure

**Figure 2:**
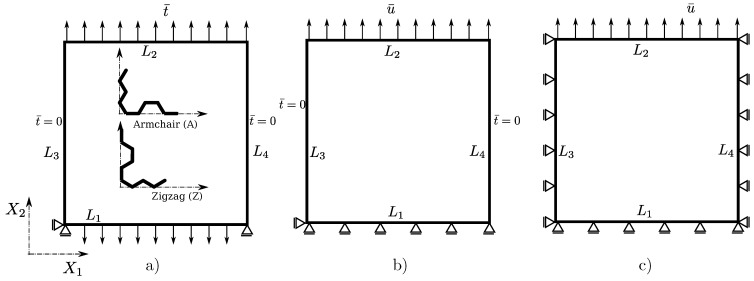
Scheme of the lattice sample with the traction (Reuss) (**a**), mixed (**b**) and displacement (Voigt) boundary conditions (BC) (**c**). The envelope of the sample is composed of lines $L_{1}$–$L_{4}$, which coincides with boundary atoms.

In this section, we present the computational procedure to perform virtual experiments, which are used to obtain the elastic properties of graphene. These are the tensile tests performed with three different choices for boundary conditions (BC) illustrated in Figure 2. In the present model, the BC are imposed atom-wise, such that either the displacement, ${\bar{d}}_{i}$, or the force, ${\bar{f}}_{i}$, is prescribed. The chosen notation, $\bar{t}$ and $\bar{u}$, is the same for the equivalent notions in continuum mechanics [30], and it is justified in the average sense.

**Figure 3:**
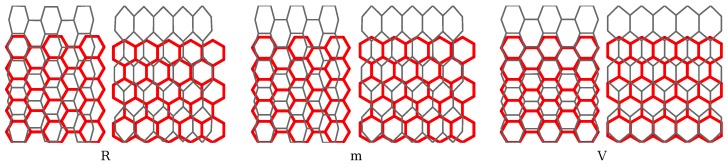
The initial and deformed shapes (scale factor 10) of the nearly square lattice of size 5 ($L_{1,2}\approx L_{3,4}$) is shown for the three types of BC. The two chiralities armchair (left) and zigzag (right) are presented for every BC case.

Let $L_{1}$ be the set of atoms that lie on the line, $L_{1}$ and, analogously, for other lines, 2–4, that form the envelope of the lattice specimen. As schematically depicted in Figure 2a, we imposed zero displacement to the minimal number of degrees of freedom (more precisely, only two atoms) in order to avoid the rigid body motion of the specimen. The force is applied to all the atoms on the lines, $L_{1}$ and $L_{2}$*i.e.*, $f_{i}=-\bar{f},\;\forall i\in L_{1}$, and $f_{i}=\bar{f},\;\forall i\in L_{2}$, while it is kept zero on remaining boundary, $f_{i}=0,\;\forall i\in \left\{L_{3},L_{4}\right\}$. The same is done for the cases shown in Figure 2b,c, with the exception of non-zero atom-wise displacement load, $d_{i}=\bar{d},\;\forall i\in L_{2}$, where the given load and displacement vectors in the $X_{1}-X_{2}$ plane (out of plane motion is not considered) are $\bar{f}={\left[0\;{\bar{f}}_{2}\right]}^{T}$ and $\bar{d}={\left[0\;{\bar{d}}_{2}\right]}^{T}$. The initial and current configuration of the nearly square shaped lattice sample for the three mentioned cases is shown in Figure 3. We use indices “R”, “m” and “V” for Reuss, mixed and Voigt type BC, respectively. The two chiralities are presented for each load/constraint case, where we call the graphene armchair or zigzag for the armchair or zigzag edges being parallel with the $X_{1}$ direction, respectively (see Figure 2a).

In the case of BC labeled as “m” and “V”, we impose the corresponding atom displacements; thus, the forces are obtained as reactions on the constrained atoms, $i,\;\forall i\in L_{2}$. Having the forces, we express the stress in standard interpretation as a force per unit of area, which differs from some previous works (e.g., [4,18,25]), where the stress is expressed per unit of length. The thickness is taken to be t=0.34 nm, corresponding to the value of interlayer distance in bulk graphite [32]. Thus, the averaged continuum stress under tension in the $X_{2}$ direction is equal to:

(24) $$\sigma_{22}=\frac{\sum_{i\in L_{2}}{\left(f_{2}\right)}_{i}}{L_{2}t}$$

where ${\left(f_{2}\right)}_{i}$ stands for given or reactive force on the atom, *i*, in load direction. For the “R” and “m” cases, we assume to have a uniaxial stress state, while for the “V” case, a biaxial state is assumed, where the average stress in the $X_{1}$ direction is analogously given as:

(25) $$\sigma_{11}=\frac{\sum_{i\in L_{3}}{\left(f_{1}\right)}_{i}}{L_{3}t}$$

where ${\left(f_{1}\right)}_{i}$ stands for reactive force on the atom, *i*, in direction, $X_{1}$. The average strain in the load direction is obtained simply as:

(26) $$\epsilon_{22}=\frac{u_{2}}{L_{3}}$$

where $u_{2}$ corresponds to given displacement, ${\bar{d}}_{2}$, for the “m” and “V” cases or to the average displacement in the “R” case. Having these results in hand, we can obtain average stiffness. The stiffness corresponding to infinitesimal deformation further provides Young’s modulus, which can be computed from the average stress and strain as follows:

(27) $${E|}_{\prime R^{\prime }\mathrm{or}\prime m^{\prime }}=\frac{\sigma_{22}}{\epsilon_{22}}{\;E|}_{\prime V^{\prime }}=\left[1-{\left(\frac{\sigma_{11}}{\sigma_{22}}\right)}^{2}\right]\frac{\sigma_{22}}{\epsilon_{22}}$$

## 3. Results and Discussion

In this section, we present numerical results for average elastic properties of SLGS under the uniaxial tensile test as a function of size, chirality and BC type using MM simulations. We show, first, the linear elastic mechanical behavior characterized by predicted Youngs modulus, with an emphasis on BC choice. The influence of the BC case is also examined in a non-linear regime, characterized by the tangential modulus value corresponding to the stress-strain relation for moderate strains. The study is concluded with detailed deformation analysis of carbon (C-C) bonds and convergence in energy, depending on the BC case. The geometry and size of the SLGS lattices used in numerical examples is depicted in Table 1.

**Table 1:** The size of the graphene lattice samples used in the numerical examples. The size parameter is used in the plots, and this table specifies the physical dimensions of the numerical test specimens.

size parameter	5	8	12	16	20	24	28
$L_{1}\left(\approx L_{3}\right)$, Å	12.03	19.26	28.89	38.52	48.15	57.78	67.41
number of atoms	66	170	350	660	984	1372	1824

### 3.1. Linear Regime and Young’s Modulus Value

**Figure 4:**
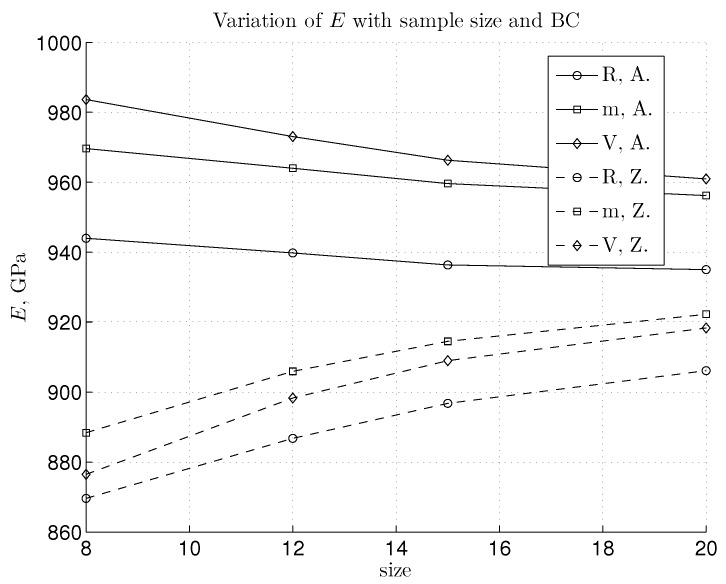
The change of Young’s modulus with respect to the size of the lattice specimen based upon the harmonic potential.

**Figure 5:**
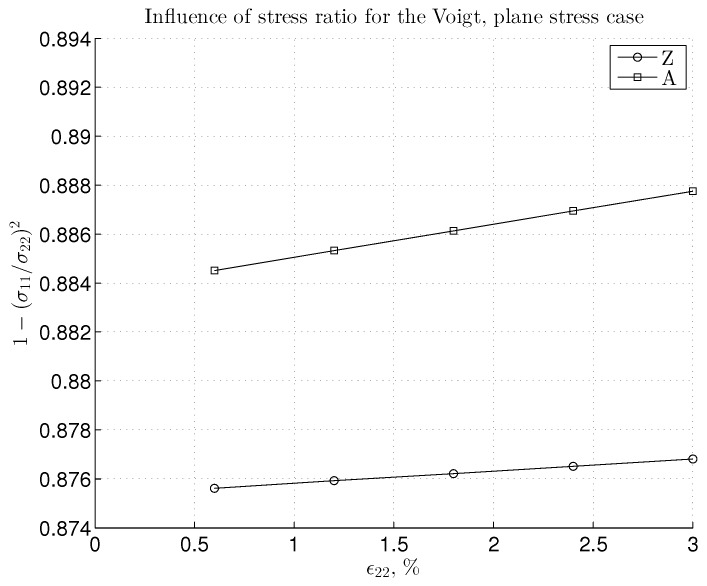
Plot of factors, including the stress ratio, that scales the expression for Young’s modulus in the plane stress state, which corresponds to the “V” BC case and a sample size of eight.

**Figure 6:**
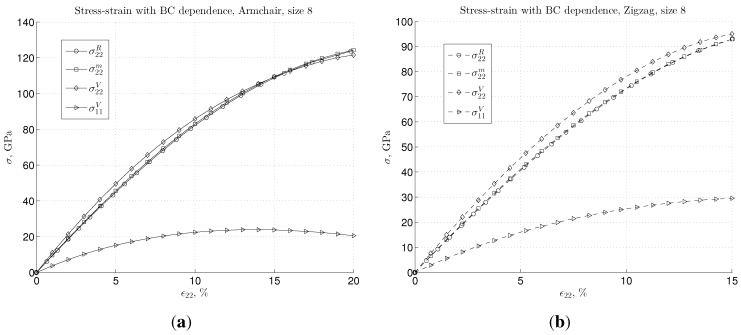
Stress-strain dependence for large strain using the Morse interatomic potential for (**a**) armchair and (**b**) zigzag graphene samples of a size of eight.

We seek to verify that the linear elastic stiffness obeys the theoretical bounds proposed in [29] and in later numerical studies [28]. In the linear regime, we can calculate Young’s modulus by using the terms in Equation (27) and harmonic interatomic potential. It is expected that the BC shown in Figure 2a would lead to the lower bound, *i.e.*, Reuss for the computed Young’s modulus, $E_{R}$. The BC in Figure 2c should give the upper bound, *i.e.*, Voigt, $E_{V}$. The response of the mixed case from Figure 2b, $E_{m}$, should be placed in-between these two bounds. While bulk graphene is considered as isotropic in a linear elastic regime (the choice made in a number of references), the true value of Young’s modulus for finite graphene depends on the edge chirality with differences between the zigzag and armchair edges. Consequently, the Youngs modulus of the finite SLGS depends on both edge chirality and size, as shown in Figure 4. In addition, we bring here the influence of the mentioned three BC types, in order to provide the best bounds for stiffness. It can be noted that a smaller zigzag sample size would influence, in general, the value of Young’s modulus more. For all BC cases and chiralities, the convergence is observed with the increase of the size of the SLGS specimen. There is no severe change in the difference between the upper and lower bound with the increase of the sample size, neither for the armchair, nor for the zigzag configuration. This difference remains rather small, $\left(E_{max}-E_{min}\right){|}_{A}\approx 40$ GPa and $\left(E_{max}-E_{min}\right){|}_{Z}\approx 20$ GPa. For larger samples (e.g., size 20) the “m” and “V” cases yield nearly the same result, giving, in this way, the upper bound. For the armchair configuration, the supposed stiffness bounds, $E_{R}\leq E_{m}\leq E_{V}$, are satisfied. On the other hand, the zigzag configuration brings, at first, a surprise, since it is a rather mixed BC, giving the upper stiffness bound. However, the normal order of bounds would be re-established without the required result post-processing to account for the plane stress conditions that occur in the “V” type BC case. Note that in Equation (27), the value of the factor with the stress ratio, $1-{\left(\sigma_{11}/\sigma_{22}\right)}^{2}$, also influences Young’s modulus (see Figure 5). From the diagrams in Figure 6, the stress ratio for the linear part (*i.e.*, for the strains lower than approximately 3%) seems to remain nearly constant. By looking more precisely at the value of the factor that includes the ratio of $\sigma_{11}/\sigma_{22}$ (depicted in Figure 5), we can explain why the zigzag configuration gives a stiffer response for the ‘m’ case. Namely, the factor that occurs in the expression, $E_{V}$, remains considerably smaller for the zigzag case, thus decreasing the value of Young’s modulus for the “V” case. This finally results in the fact that the “m” case yields the upper bound in the linear regime.

### 3.2. Nonlinear Regime and Tangential Modulus

We further discuss how the three BC cases would influence the stiffness bounds in the nonlinear regime for the case of moderate strain. In such a case, we must employ the modified Morse potential, as described in Section 2.2. Here, we compute the average continuum stress with respect to the initial configuration, as defined in Equation (24) and Equation (25), along with the nominal measure for strains given in Equation (26). These results are plotted in Figure 6 for both chiralities in terms of stress-strain diagrams for a nonlinear regime.

A number of interesting observations can be made from these stress-strain plots. As shown in Figure 6a, for the armchair graphene, the stress-strain dependence shows similar behavior as for the small strain case, up to the strain, $\epsilon_{22}$, around 15%. Namely, stress in the load direction again respects relation $\sigma_{22}^{V}>\sigma_{22}^{m}>\sigma_{22}^{R}$, whereas for the Reuss and mixed cases, the difference remains negligible. For the strain, $\epsilon_{22}\approx 15\%$, as transversal stress, $\sigma_{11}$, stops increasing, *i.e.*, it reaches its maximum, the difference between the stress of upper bound, “V”, and the lower one, “m” and “R”, becomes negligible. Furthermore, for even larger strains ($\epsilon_{22}>15\%$), the order of the bounds is changed, *i.e.*, the relation becomes $\sigma_{22}^{V}<\sigma_{22}^{m,R}$. Note that for the zigzag configuration, we presented a stress-strain diagram in the strain range, 0–15%. This is because increasing strain slightly causes the C-C, pair bond separation to come to the point where brittle failure occurs; see e.g., [32,35]. In zigzag configuration, approximately one third of the C-C bonds are parallel with the load and, thus, are more strained; see Section 3.3 for a detailed discussion. The questions of bond breakage belong to issues of quantum chemistry and a more complex description of atom interaction, and thus, it will not be discussed herein. However, for the zigzag graphene in the presented strain range, the transversal stress, $\sigma_{11}$, does not reach its maximum. Consequently, the difference in stress for “V” and “m” (or “R”) is noticeable throughout, as depicted in Figure 6b.

**Figure 7:**
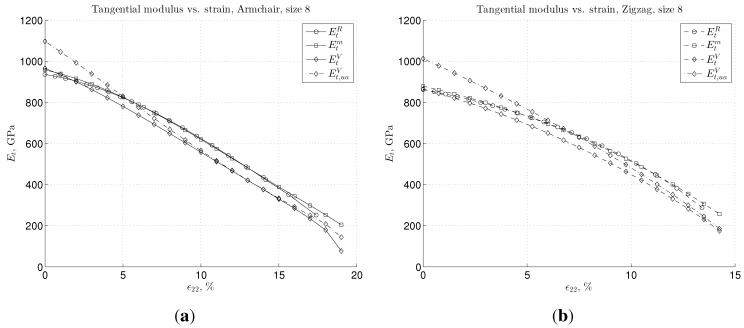
Tangential modulus-strain dependence for large strain using the Morse interaction shown for: (**a**) armchair and (**b**) zigzag graphene sample of the size of eight.

The plots showing the tangential modulus *vs*. strain are given in Figure 7, for both chiralities and for the three given types of BC. In Figure 7, we also show the tangential modulus for the “V” case calculated from the averaged stress and strain by using the expression for the uniaxial stress state, denoted as $E_{t,ua}^{V}$, see Equation (27). Note that for the infinitesimal strain, this leads to Young’s modulus ($E_{t}\mapsto E$), which is overestimated by more than 100 GPa. This could be one of the main reasons for the scatter of the previously available results, as mentioned in the Introduction. However, for strains, $\epsilon_{22}$, larger than approximately 5%, this difference of treating the “V” case as uniaxial or biaxial becomes negligible. We also note that in the nonlinear regime, the tangential stiffness shows the lowest values for the “V” BC case, for both armchair and zigzag configuration. This is due to the plane stress state modification used for this BC case.

### 3.3. Energy and Deformation of Bonds

**Figure 8:**
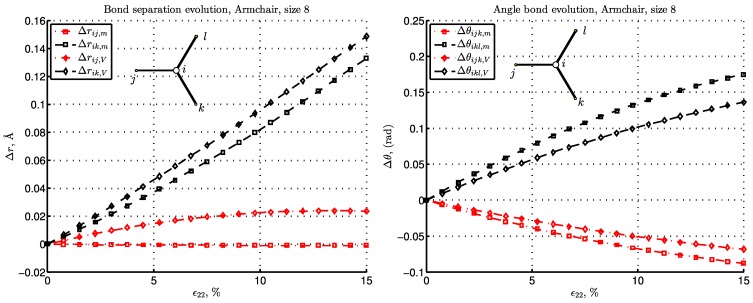
The pair bond separation (left) and angular bond (right) evolution with respect to strain increase for armchair graphene.

**Figure 9:**
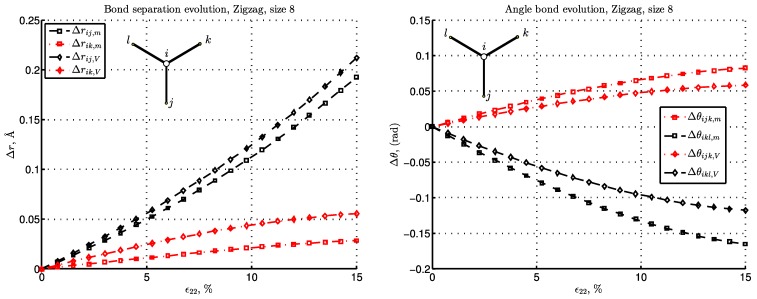
The pair bond separation (left) and angular bond (right) evolution with respect to strain increase is presented for zigzag graphene.

We further carry out the energy and the deformation studies of the lattice network. This can be performed for a typical pattern of hexagonal microstructure for armchair and zigzag graphene, presented in Figure 3 for the aforementioned BC types. We will first present the deformation in C-C bonds by picking up the bulk atom *i*, as shown in Figure 8 and Figure 9. Note that the bulk atom denotes any atom that is far enough from the boundary. The computation is performed for the pair bond separation, $\Delta r=r-r_{0}$, and angular bond evolution, $\Delta \theta =\theta -\theta_{0}$, for the given load increase. The terms, Δr and Δθ, govern the energy evolution of the system, as shown in Equation (8) and Equation (9). Since the difference between the BC types “R” and “m” is negligible in the presented strain range, the Reuss BC is further omitted. Due to symmetry, for the bulk atoms, the bond separations evolution, $\Delta r_{ik}$, is equal to $\Delta r_{il}$, as well as bond angles, $\Delta \theta_{ijk}=\Delta \theta_{ijl}$. Thus, $\Delta r_{il}$ and $\Delta \theta_{ijl}$ are omitted, as well.

For the armchair configuration in Figure 8 with the bond, i-j, perpendicular to the loading direction, we note the following. For the case, “m”, the separation, $\Delta r_{ij}$, is negligible for small strain and becomes negative as the strain increases, thus yielding some compression for moderate strain. For the “V”-type BC that constrains the lateral contraction, this bond is stretched. Note that the bonds orthogonal to the load direction, like i-j in armchair graphene, are dominant in forming the average lateral stress, $\sigma_{11}$, which explains, also, the resemblance to the $\sigma_{11}^{V}$ curve presented in Figure 6a. Note also that for the “V” case, pair bonds are significantly more strained than in the “m” case, while angular bonds, on the other hand, are less strained.

What is specific for the zigzag configuration is that one third of the bonds, like i-j in Figure 9, is parallel to the load. Thus, in this configuration, the bond stretch, Δr, is nearly double the one in the armchair configuration, as can be seen by comparing the left plots in Figure 8 and Figure 9. In the zigzag configuration, there is, consequently, no perpendicular bond, nor a bond whose deformation is negligible. The angle change, Δθ, shows analogous behavior for the cases, “m” and “V”, as stated above for the armchair lattice.

**Figure 10:**
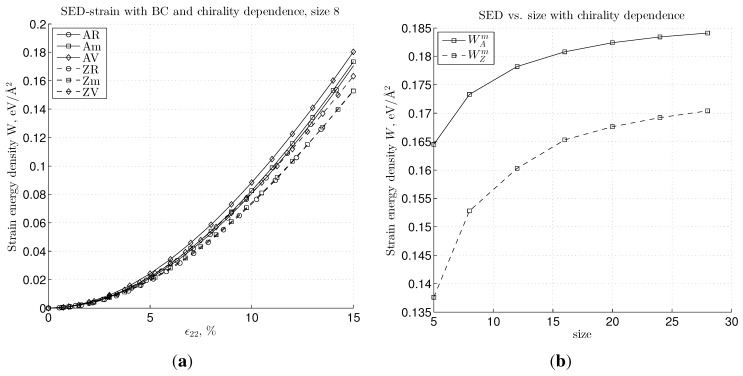
The strain energy density plot shows the dependence on the chirality (armchair and zigzag) and BC types, “R”, “m” and “V”, in (**a**) and the influence of size and chirality to the strain energy density in (**b**) (for the “m” BC case and strain $\epsilon_{22}=15\%$).

The influence of the BC on the strain energy density, *W*, is presented in Figure 10. First, we picture the *W vs*. strain relation in Figure 10a, which shows the relation, $W_{R}\leq W_{m}\leq W_{V}$, for both chiralities; however, the zigzag configuration yields lower energy than the armchair. Note also that these differences in the calculated strain energy density become more pronounced in the moderate strain regime. The convergence of the strain energy can be seen in Figure 10b, where *W* *vs*. size is plotted with the chirality parameter for the “m” BC case. An increase in sample size corresponds to a decrease of the fraction of boundary atoms with respect to the bulk atoms, which then leads to the convergence of the strain energy.

## 4. Conclusions

The key question addressed in this work pertains to an explanation of the very wide scatter of reported results on the elastic properties of graphene. Our study points out one of the key factors for this kind of scatter in Young’s modulus as caused by different types of BC, with values of around 40 and 20 GPa for armchair and zigzag configuration, respectively. For the Voight-type BC with a given displacement imposed over the four edges forming the envelope of the graphene sheet, this scatter rises up to more than 100 GPa, if we do not impose the constraint corresponding to the plane stress condition.

It has also be found out that the standard linear stiffness bounds hold for the armchair configuration, while for the zigzag configuration, they do not. Moreover, for the non-linear regime with moderate strain of the lattice, the stiffness bounds do not apply. Furthermore, the difference in the computed results for the tangential modulus for three given BC types is significantly larger then for Young’s modulus (around 100 GPa).

Through the study of intearatomic bond structure, the corresponding deformation and energy, we can confirm the importance of the type of the BC imposed on the lattice. This is certainly one of the main sources for the computed response discrepancy that is typical in the currently available literature.
